# Plant Resistance to Fungal Pathogens: Bibliometric Analysis and Visualization

**DOI:** 10.3390/toxics10100624

**Published:** 2022-10-19

**Authors:** Yueyue Tang, Guandi He, Yeqing He, Tengbing He

**Affiliations:** 1 College of Agriculture, Guizhou University, Guiyang 550025, China; 2 New Rural Development Research Institute, Guizhou University, Guiyang 550025, China

**Keywords:** Web of Science, fungal pathogens, plant resistance, diseases

## Abstract

Plants are susceptible to fungal pathogen infection, threatening plant growth and development. Researchers worldwide have conducted extensive studies to address this issue and have published numerous articles on the subject, but they lack a scientometric evaluation. This study analyzed international research on the topic “Plant resistance to fungal pathogens” between 2008 and 2021, using the core database of the Web of Science (WoS). By searching the subject words “Plants”, “Disease Resistance”, and “Fungal Pathogens”, we received 6687 articles. Bibliometric visualization software analyzes the most published countries, institutions, journals, authors, the most cited articles, and the most common keywords. The results show that the number of articles in the database has increased year by year, with the United States and China occupying the core positions, accounting for 46.16% of the total published articles worldwide. The United States Department of Agriculture (USDA) is the main publishing organization. Wang Guoliang is the author with the most published articles, and the Frontiers in Plant Science ranks first in published articles. The research on plant anti-fungal pathogens is booming, and international exchanges and cooperation need to be further strengthened. This paper summarizes five possible research ideas, from fungal pathogens, gene editing technology, extraction of secondary metabolites from plants as anti-fungal agents, identification of related signal pathways, fungal molecular databases, and development of nanomaterials, to provide data for related research.

## 1. Introduction

There are millions of different types of fungal infections, many of which represent a serious threat to plant health. Although long recognized, fungi were not acknowledged as the cause of plant diseases until the 1850s [1]. Cranberry blight caused by *Stagonosporopsis cucurbitacearum* [2,3], blueberry grey mold produced by *Botrytis cinerea*, streak disease caused by *Drechslera avenaceous* [4], and leaf anthracnose of tea plants caused by the *Colletotrichum* [5] are some examples of diseases and pathogens feared as much as human diseases and war.

Fungal pathogens colonize plants in different ways to obtain nutrients. Biotrophic fungi feed only in living plant tissues. They do not kill the host, preventing cell death and manipulating plant metabolism by secreting effector molecules. Necrotrophic fungi infect living tissues, continuously produce hydrolytic enzymes, and secrete toxins to kill plant cells and acquire nutrients [6]. Hemibiotrophs initially extract nutrients from living tissues before switching to a necrotrophic phase.

Plants cannot move like animals and are more likely to be infected by multiple fungal pathogens. In response to the stress of fungal pathogens, plants resist the invasion of fungal pathogens through innate or acquired systemic immunity. For example, fungal pathogens invade the cell wall, the first line of defense of the plant host, by autophagy of plant cells against necrotrophic fungal pathogens [7], secretion of phytohormones, such as salicylic acid (S.A.), jasmonic acid (J.A.), and ethylene (E.T.) involved in regulating resistance to fungal pathogens [8], and secretion of secondary metabolites [9]. However, many fungi do not require hosts. Some fungi can even take multiple plants as hosts due to the low level of plant resistance and infectious pathogens such as viruses. Additionally, fungi are extremely dynamic and can produce distinct sporophores and spores to facilitate rapid spread [10]. Around the world, many plants are suffering huge losses due to fungal infections. In the 19th century, an outbreak of *Phytophthora*, the causative agent of potato late blight, led to a great famine in Ireland [11]. Ash dieback, which appeared in Poland in the 1990s, rapidly spread to most Eastern, Central, and Northern European countries, posing a fatal threat to ash trees in temperate regions of Europe [12]. Intensive agricultural production practices make plants more susceptible to fungus-related pathogens. According to harvest statistics for the world’s five most important crops (rice, wheat, maize, potatoes, and soybeans), the annual losses caused by fungi were enough to meet the crop needs of 595 million people in 2009–2010 [13].

In the past two decades, many studies have been conducted by domestic and foreign researchers to reduce the losses caused by plant infections with fungal pathogens. However, there is a lack of a thorough and impartial bibliometric examination. British intelligence scientist A. Pritchard developed bibliometric analysis, which offers the advantages of objectivity, quantification, and adaptability. It is an important method for studying the characteristics and content of documents and is widely used in dynamic research and analysis in different disciplines [14]. This paper examines the published articles on plant resistance to fungal pathogens in the Web of Science (WoS) core database from 2008 to 2021, identifies major countries, journals, and authors using various metrology approaches, and evaluates the primary research content over time. The findings may provide very important recommendations for future research on this topic.

## 2. Materials and Methods

### 2.1. Data Collection

This study focuses on the scientometric analysis of plant resistance to fungal pathogens from 2008 to 2021. All the literature data are from the Clarivate Analytics WoS core collection database (https://www.webofscience.com/wos/woscc/basic-search, accessed on 18 October 2022). The data retrieval time is 27 September 2022. The following keywords are entered to search: “plant”, “disease resistance”, and “fungal pathogen”, excluding conference papers, book chapters, duplicate literature, etc. A total of 6687 papers are retrieved for further study after collecting and screening domestic and international literature on plant resistance to fungal pathogens research.

### 2.2. Data Analysis

We used VOSviewer to map the network collaboration of major countries, institutions, authors, journals, etc. Bibliometrics obtained the number of papers published in different years in some countries; the keywords of CiteSpace can also well reflect the keywords in different periods. HistCite created a chronological chart of the top 50 local citation scores (LCS).

VOSviewer is a software developed by the Science and Technology Research Center of Leiden University in the Netherlands. It can draw knowledge maps through the relationship construction and visual analysis of “network data” and visualize the relationship between the structure, evolution, and cooperation of the field [15]. Thomson Reuters developed HistCite, which can only be applied to WoS database files owned by the same company. This paper understands the domestic and foreign attention to this field by analyzing the annual publication volume, major countries, institutions, authors, journals, and other network cooperation maps. Next, we analyzed the keywords in this field to understand the research hotspots and then analyzed the main research in this direction.

## 3. Results

### 3.1. Citation Analysis

**Figure 1:**
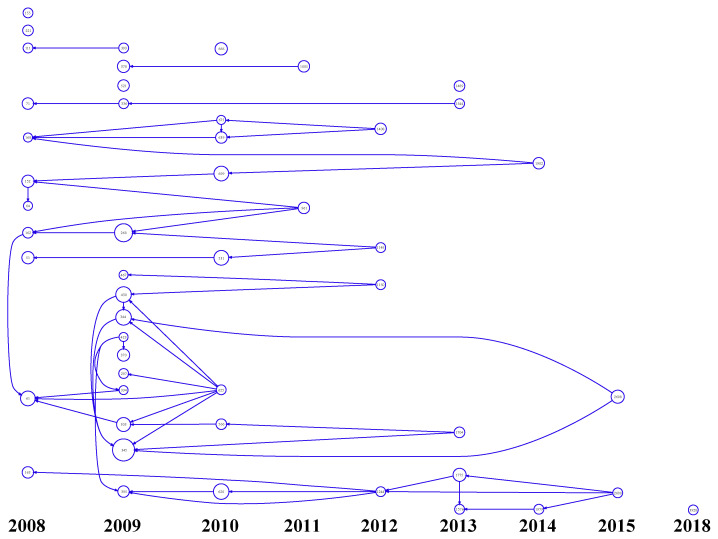
The first 50 papers on plant resistance to fungal pathogen based on the local citation score (LCS). The figure in the circle indicates the literature number, and the diameter of the circle is proportional to the LCS.

LCS is used as a bibliometric criterion to analyze the searched articles, and shows that the top 50 articles are concentrated in 2008–2015 (Figure 1). The literature with the numbers 345 [16] and 268 [17] were the most cited.

**Figure 2:**
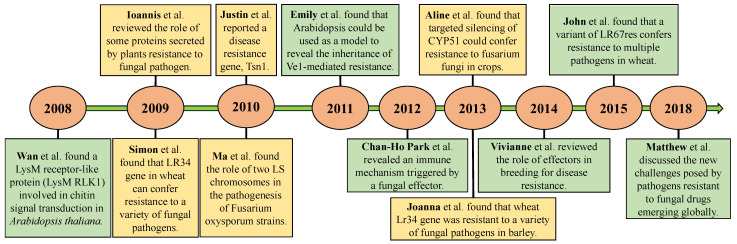
Milestone publications on plant resistance to fungal pathogens during 2008–2018. Numbers in circles represent years. These important research findings included Wan et al. [18], Ioannis et al. [17], Simon et al. [16], Justin et al. [22], Ma et al. [19], Emily et al. [20], Chan-Ho Park et al. [23], Aline et al. [24], Joanna et al. [25], Vivianne et al. [26], John et al. [27], and Matthew et al. [21].

The examination of the first 50 publications classified according to LCS index gave us an indication on the progress of research and the keystone in the field of resistance of the plant to fungal pathogens (Figure 2). In 2008, Wan et al. [18], the authors of a paper numbered 61 with an LCS of 75, identified a LysM receptor-like protein required for chitin signaling in *Arabidopsis thaliana*. In 2009, Simon et al. [16], in the article numbered 345 published in SCIENCE, proposed the wheat gene Lr34 useful to induce resistance to some fungal pathogens. In 2010, studies on the molecular basis of Fusarium pathogenicity were published in NATURE [19]. In 2011, the article with the highest LCS was published in PLANT PHYSIOLOGY [20] and developed an RLP-mediated verticillium wilt resistance signaling model in *A. thaliana* by transferring the tomato gene encoding Ve1. Matthew et al. [21] reviewed the studies on plant resistance to fungal pathogens, underlined the emergence of plant pathogenic fungi resistant to antifungal drugs, and showed several problems in this field that need to be solved.

### 3.2. Annual Research Analysis

**Figure 3:**
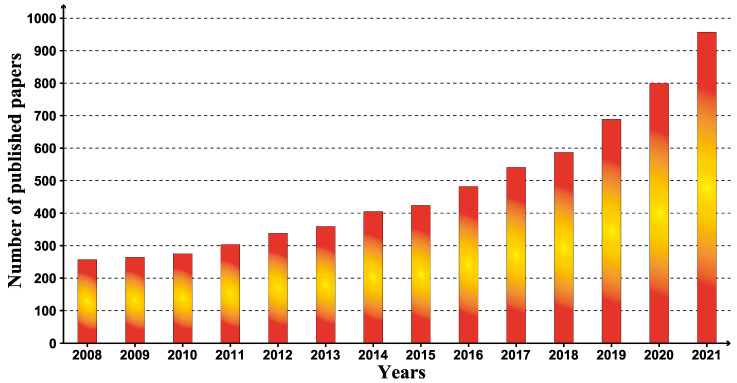
Annual trend of papers on plant resistance to fungal pathogens from 2008 to 2021. The data were retrieved from the Web of Science databases.

The literature on plant resistance to fungi infections is primarily focused on the years 2008–2021 in the WoS core collection, and the number of papers published after 2008 is rising annually. Figure 3 demonstrates that, as of 2021, the number of articles relating to research on anti-fungal pathogens is continually increasing, growing substantially from 257 in 2008 to 962 in 2021, suggesting that this study area has attracted widespread interest since 2008. There is still great room for progress in the future with increasing attention.

### 3.3. Research Analysis

#### 3.3.1. Countries Assessment

Articles published by countries included in the WoS database can reflect the research level and importance of countries in this field to a certain extent. The data obtained in this study shows that the sources of articles in the WoS database include 124 countries (for details see Supplementary Table S1), and Table 1 lists the top ten countries, namely the United States, China, Germany, India, Australia, the United Kingdom, France, Canada, Brazil, and Italy, accounting for the 93.48% of the total publication volume in the field. The United States and China issued far more documents than third-ranked Germany. This partially reflects that the two countries have paid more attention to plant resistance to fungal pathogens. The local total citation score (TLCS) and the global total citation score (TGCS) can more scientifically reflect their corresponding academic influence collectively. Table 1 shows that the TLCS and TGCS of the United States are larger than those in other countries.

**Figure 4:**
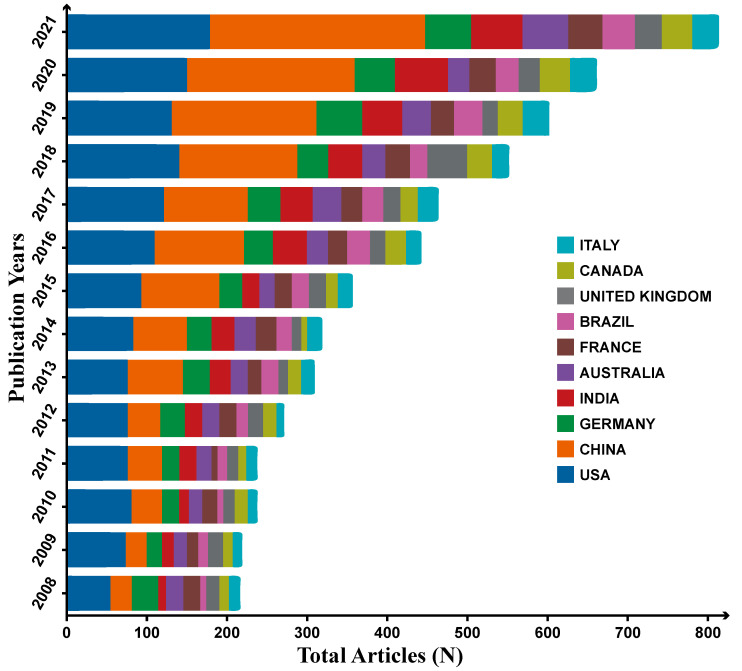
The first ten countries were selected based on the publications included in the Web of Science database.

We analyzed the annual publication volume of the top ten countries by publication volume, as shown in Figure 4. From 2008 to 2012, the United States published the most documents, while from 2013 to 2018, the United States and China published the same number of documents. From 2019 to 2021, China had the largest number of documents, followed by the United States. From 2008 to 2021, except for China and the United States, the ranking of the number of published papers has changed, and the ranking of other countries has not changed much. However, each country’s published papers have increased yearly, indicating research on plant resistance to fungal pathogens. The field is getting more and more attention from the world.

**Figure 5:**
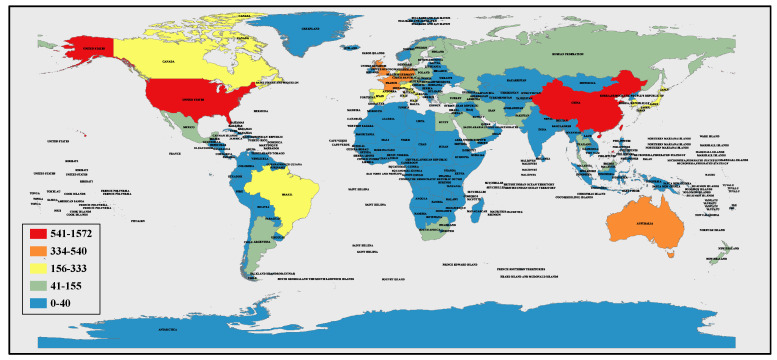
Distribution chart of the number of papers published in the field of plant fungal pathogen resistance in the period 2008–2021 around the world. The color scale is illustrated on the left.

The distribution of articles published on plant resistance to fungal pathogens in the period 2008–2021 and here analyzed are shown in Figure 5. The United States, China, Canada, and a few other countries are the main research forces.

#### 3.3.2. Country Collaboration

**Figure 6:**
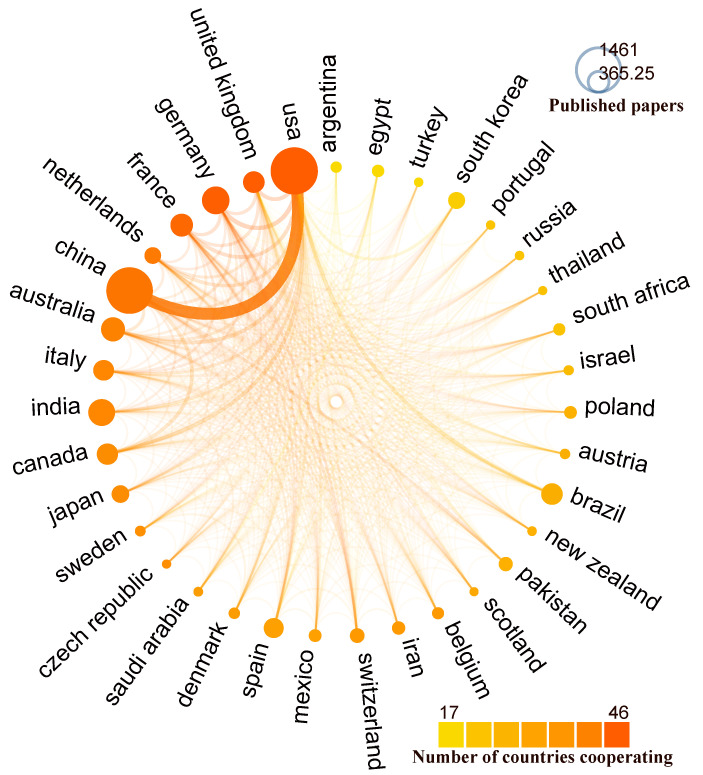
Country collaboration map. Each country is represented by a circle; the size of the circle varying with the total number of publications produced and the connection lines between two or more countries represent the strength of collaboration between different countries.

Figure 6 shows a map of cooperation between some countries. It can be seen from the Figure that, except for the close cooperation between the United States and China, and the relatively close cooperation between the United States and Brazil, among the United Kingdom, France, Germany, and Canada there are cooperative relations between countries, but they are not very close.

#### 3.3.3. Institutions Analysis

The articles on plant anti-fungal pathogens included in the WoS database involved a total of 4987 institutions (for information on the remaining institutions, see Table S2 in Supplementary Materials). The top ten are listed in Table 2. The institutions that published the most articles were the USDA from the United States (251 articles) and China (231 articles). Although the number of papers published by these two institutions is not much different, the TLCS and TGCS of the USDA are significantly higher than those of Chinese Acad Agr Sci. Six of the top ten institutions are from China, three are from the United States, and one is from Canada.

**Figure 7:**
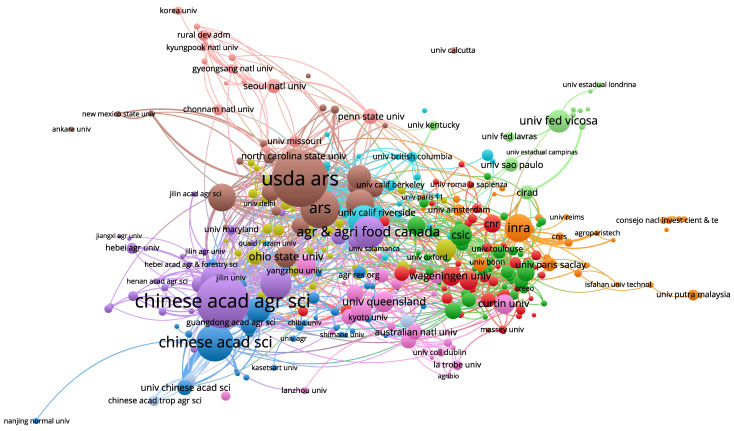
Institutional collaboration map. There are 643 circles and 5704 lines. The size of the circle is proportional to the number of documents published by the organization, and the thickness of the line is proportional to the partnership between the organizations.

Analysis and research institutions can quickly find teams with greater scientific influence in the field, providing quick references for data acquisition and cooperation. In general, the cooperation and exchanges between institutions are relatively close, and the institutions that publish more articles have closer exchanges with other institutions (Figure 7).

#### 3.3.4. Author Analysis

**Figure 8:**
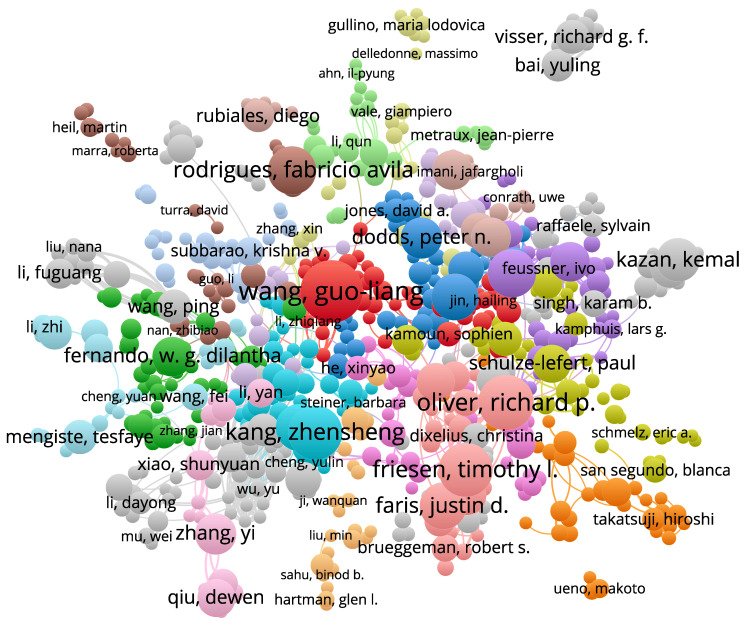
A network diagram of author collaborations on plant resistance to fungal pathogens. With 28,507 nodes and 3006 connections, the size of the circle is proportional to the number of articles published by the author. The thickness of the lines is proportional to the closeness of the collaboration between the authors.

Figure 8 shows the network relationship graph of author cooperation and co-occurrence involved in studies on plant resistance to fungal pathogens (detailed author information is provided in Supplementary Table S4). Wang Guoliang, Oliver Richard p, Friesen, etc., are the most important researchers. In addition, our data show that many researchers are engaged in this research and have a cooperative relationship.

### 3.4. Journal Analysis

Table 3 shows the top ten journals in terms of publishing volume (see Supplementary Table S3 for complete information), their impact factor (IF), country of publication, and total citations. As can be seen from Table 3, the top three journals with the number of published articles are Frontiers in Plant Science published in Switzerland (300 articles), European Journal of Plant published in the Netherlands (205 articles), and Plant Disease published in the United States (175 articles). Four top ten journals are published in the U.S, three in the U.K, and the rest in Switzerland, the Netherlands, and Germany. The IF is between 1.91–5.75, and the total number of citations is 6146 times at the highest and 2018 times at the lowest.

### 3.5. Keyword Analysis

**Figure 9:**
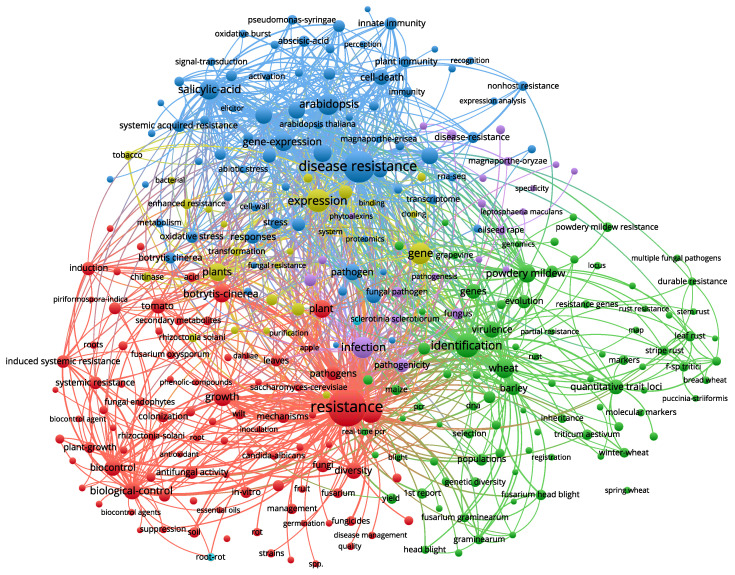
Keyword network visualization. The size of the circle is proportional to the number of keyword occurrences. Frequency of the top 20 keywords used in the publications included in the Web of Science database.

Figure 9 and Table 4 shows a network co-occurrence diagram of plant resistance to fungal pathogens (details are provided in Supplementary Table S5). According to the statistics, the most often appearing terms from 2008 to 2021 are resistance, disease resistance, identification, expression, disease, *Arabidopsis*, genes, infection, S.A., and wheat.

**Figure 10:**
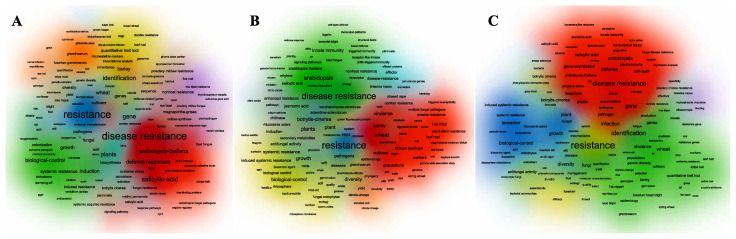
Visualization of keywords networks at different stages. (**A**) 2008–2012, (**B**) 2013–2018, (**C**) 2019–2021.

We conducted visual analysis in the three time periods of: 2008–2012, 2013–2018, and 2019–2021, to fully comprehend the dynamic changes of keywords. We discovered that biological control was a prominent topic, and that resistance and disease resistance were not the only key phrases that persisted in these three periods with the highest frequency (Figure 10). From 2008 to 2018, powdery mildew was a relatively hot topic, but from 2019 onwards, its popularity dropped significantly. Wheat was the main research object in 2008–2012, and the research interest in 2013–2018 dropped significantly, but the research interest in 2019–2021 is also the word gene. As can be seen from the graph, research on *Arabidopsis* has increased significantly since 2013.

To determine the development of a research frontier, we used CiteSpace’s burstness feature. The top 20 keywords with the most powerful citation bursts are displayed in Table 5. The Table shows that most of the keywords are largely focused on 2008–2012 and 2019–2021. The highest intensity is *Magnaporthe grisea*, systemic acquired resistance, and hypersensitive response. The intensity is 15.7, 12.88, and 12.41, respectively.

## 4. Discussion

Fungal pathogens are one of the most important factors affecting plant growth and development and threaten food security [13]. The bibliometrics analysis can accurately understand the research background and forecast future research prospects. The primary purpose of this study is to perform a quantitative analysis of 6687 articles published on plant resistance to fungal pathogens included in the WoS database from 2008 to 2021 to identify some research trends and provide references for future researchers.

According to the annual publication volume, the publication volume of this research is continuously increasing year after year, attaining significant growth from zero to even more. The first article on plant resistance to fungal pathogens included in the WoS database was published in ORGANIC LETTERS in 1999, in which the authors described the metabolism and detoxification of destruction and Homodestruxin B by oil crops [28]. In addition, in 2003, PHYTOCHEMISTRY published a second related article on the antitoxin produced by cress under biotic stress [29], and the authors of the first two articles were Pedras. In 2008, Wan et al. pointed out that chitin is a component of fungal cell walls, which can be sensed by plant cells to induce plant cell immunity. They found the receptor protein LysM RLK1 required for chitin signaling in *Arabidopsis thaliana* [18]; PEN1 syntaxin is a component of barley against *Ascomycete* powdery mildew fungi [30]. Fukuoka et al. (2009) discovered a gene that could improve rice blast resistance, and the pi21 allele had the same effect on some japonica rice lines [31]. Chen et al. (2010) identified a new class of sugar transporters, SWEETs, which are critical for plant growth and development, and whose expression can be targeted by fungal pathogens for nutrient acquisition [32]. According to Armin et al. (2011), plant cells can absorb and disseminate the fungal chorismate mutase released by the fungus that causes corn smut, altering the metabolic state of these cells through metabolic initiation [33]. Chen et al. (2014) proposed the mechanism of graphene (G.O.) inhibiting fungal pathogens and believed that G.O. might have anti-fungal activity against multidrug-resistant fungal pathogens [34]. Wang et al. (2016) used tomato and *Arabidopsis thaliana* as experimental materials to target the silencing of the Bc-DCL gene to attenuate the pathogenicity of fungal pathogens [35]. Zhang et al. (2017) obtained the mutant wheat plant Taedr1 by CRISPR/Cas9 technology and found that the plant could resist powdery mildew [36]. Su et al. (2019) found that a gene (HRC) is a decisive factor in resistance to *Fusarium* head blight (FHB), and the TaHRC sequence can be manipulated by bioengineering methods to improve FHB resistance of other cereal crops [37]; The UNITE database fungal ITS sequences that can identify fungi have increased and become more powerful [38]. Luo et al. (2021) tried introducing five resistance genes into wheat, four of which were functional, indicating that it was feasible to introduce multiple genes to improve plant durability and spectral resistance [39].

There is a difference of 55 articles between the United States and China, but a difference of 22,134 times in TGCS (Table 1); a difference of 20 articles between the U.S. USDA and Chinese Acad Agr Sci in China, but a difference of 2776 times in TGCS (Table 2); four belonged to the United States and none to China (Table 3). These results indicate that the United States has the most prominent contribution and influence on plant anti-fungal pathogens, followed by China. The national and institutional cooperation network map analysis shows that they all have cooperative relations. For example, the most comprehensive UNITE database of fungi identification is jointly established by Sweden, the United Kingdom, the United States, Germany, and other countries. UNITE is the most commonly used database for comparing fungal taxonomy and annotation after ITS high-throughput sequencing [38].

Visual analysis of keywords shows that the research content of researchers is also more diversified. Due to the long time required for traditional crossbreeding to select virus-resistant varieties, chemical fungicide drug residues will cause serious environmental pollution [40]. Therefore, exploring more effective methods to solve this problem is necessary. Through the analysis of keywords, the current main research can be classified into the following five categories: (1) At the molecular level, research on the transmission route, reproduction mode, regulatory mechanism, and interaction mechanism between virus and host of fungal pathogens, disease resistance genes, and susceptibility diseases, gene mapping, using gene editing technology to target knockout or overexpression of key genes to obtain varieties with strong disease resistance; (2) Extract secondary metabolites secreted by plants with anti-fungal pathogens to make anti-fungal agents for protection, plants are protected from infection by some fungal pathogens; (3) Identify the signaling pathways of fungal pathogens infecting plant hosts, and edit key regulatory factors upstream of the signaling pathways to avoid infection of plant hosts by fungal pathogens; (4) Further improve the established fungal molecular identification database, such as UNITE database (https://unite.ut.ee/, accessed on 18 October 2022) [38], or establish a fungal pathogen identification database; (5) Develop metal nanomaterials to make pesticides and nano herbicides and realize the combination of green chemistry and nanobiotechnology.

## 5. Conclusions

Our research shows that articles on plant anti-fungal pathogens are increasing each year. Through bibliometric analysis of the articles included in WoS from 2008 to 2021, the following conclusions are drawn: (1) The top three countries in terms of publication volume are the United States, China, and Germany; among the ten institutions, six are in China, three in the United States, and one in Canada. The top ten journals are from the United States, the United Kingdom, Switzerland, the Netherlands, and Germany; the United States has the strongest comprehensive influence, followed by China; (2) The cooperative relationship between different countries needs to be further strengthened; (3) The systematic omics and comparative omics (informatization) databases should be integrated to facilitate collaborative research; (4) The research has a significant interdisciplinary nature. There are still a lot of issues and challenges in the current research that need to be further solved.

## Figures and Tables

**Table 1:** Global research trends 2008–2021 by publications included in the Web of Science database, total local citation score (TLCS) and total global citation score (TGCS).

Country	Documents	Proportion (%)	TLCS	TGCS
USA	1571	23.493	4263	56,964
China	1516	22.671	2749	34,830
Germany	541	8.090	1849	21,892
India	517	7.731	852	12,487
Australia	421	6.296	2093	17,568
UK	374	5.593	1346	18,974
France	372	5.563	1128	14,456
Canada	334	4.995	811	10,901
Brazil	315	4.711	339	6714
Italy	290	4.337	698	8957

TLCS refers to the number of local citations; TGCS refers to the number of citations, which is the number of citations displayed in the WoS database.

**Table 2:** The first ten institutions by published research papers on plant resistance to fungal pathogens included in the Web of Science database, total local citation score (TLCS), and total global citation score (TGCS).

Institution	Country	Documents	Proportion (%)	TLCS	TGCS
USDA	USA	251	3.754	723	8349
Chinese Acad Agr Sci	China	231	3.454	544	5573
ARS	USA	149	2.228	464	5007
Chinese Acad Sci	China	140	2.094	328	5033
Nanjing Agr Univ	China	120	1.795	194	2915
Northwest A&F Univ	China	115	1.720	125	2154
Agr and Agri Food Canada	Canada	113	1.690	420	4614
Univ Calif Davis	USA	113	1.690	381	5469
Huazhong Agr Univ	China	110	1.645	381	4165
China Agr Univ	China	100	1.495	215	2674

**Table 3:** The most influential journals included in the Web of Science database related to the number of publications, citations, and 2021 impact factor (IF) during 2008–2021 in the field of plant resistance to fungal pathogens.

Journal	Country	Documents	Citations	IF (2021)
Frontiers in Plant Science	Switzerland	300	6146	5.75
European Journal of Plant Pathology	Netherlands	205	3577	1.91
Plant Disease	USA	175	2018	4.44
Plos One	USA	164	5024	3.24
Molecular Plant Pathology	Britain	144	5381	5.66
Phytopathology	USA	143	2780	4.03
Plant Pathology	Britain	137	2390	5.59
Molecular Plant-microbe Interactions	USA	127	5101	4.17
Scientific Reports	Britain	116	2571	4.38
Theoretical and Applied Genetics	Germany	110	2883	5.70

**Table 4:** Top 20 keywords.

NO.	Keyword	Occurrences	NO.	Keyword	Occurrences
1	Resistance	1888	11	Defense	416
2	Disease Resistance	1308	12	Gene-expression	405
3	Identification	724	13	Growth	388
4	Expression	637	14	Plants	371
5	Disease	547	15	Powdery-mildew	370
6	Arabidopsis	538	16	Plant	363
7	Gene	537	17	Biological-control	349
8	Infection	533	18	Defense-Responses	338
9	Salicylic-acid	480	19	Arabidopsis-thaliana	336
10	Wheat	430	20	Botrytis-cinerea	329

**Table 5:** Top 20 keywords with strongest citation bursts. The strength represents the number of times the keyword appears.

Outbreak Word	Year	Score	Start	End	2021–2003
*Magnaporthe grisea*	2003	15.70	2008	2012	▂▂▂▂▂ ▃▃▃▃▃ ▂▂▂▂▂▂▂▂▂▂▂▂▂▂
Systemic acquired resistance	2003	12.88	2008	2012	▂▂▂▂▂ ▃▃▃▃▃ ▂▂▂▂▂▂▂▂▂▂▂▂▂▂
Induction	2003	9.83	2008	2011	▂▂▂▂▂ ▃▃▃▃ ▂▂▂▂▂▂▂▂▂▂▂▂▂▂▂
Salicylic acid	2003	8.93	2008	2009	▂▂▂▂▂ ▃▃ ▂▂▂▂▂▂▂▂▂▂▂▂▂▂▂▂▂
Chitinase	2003	8.23	2008	2014	▂▂▂▂▂ ▃▃▃▃▃▃▃ ▂▂▂▂▂▂▂▂▂▂▂▂
Hypersensitive response	2003	12.41	2009	2014	▂▂▂▂▂▂ ▃▃▃▃▃▃ ▂▂▂▂▂▂▂▂▂▂▂▂
*Magnaporthe oryzae*	2003	9.33	2009	2013	▂▂▂▂▂▂ ▃▃▃▃▃ ▂▂▂▂▂▂▂▂▂▂▂▂▂
Maize	2003	9.86	2010	2014	▂▂▂▂▂▂▂ ▃▃▃▃▃ ▂▂▂▂▂▂▂▂▂▂▂▂
Antifungal protein	2003	9.16	2010	2014	▂▂▂▂▂▂▂ ▃▃▃▃▃ ▂▂▂▂▂▂▂▂▂▂▂▂
Tobacco	2003	10.70	2011	2014	▂▂▂▂▂▂▂▂ ▃▃▃▃ ▂▂▂▂▂▂▂▂▂▂▂▂
Flax rust	2003	8.94	2011	2014	▂▂▂▂▂▂▂▂ ▃▃▃▃ ▂▂▂▂▂▂▂▂▂▂▂▂
Abscisic acid	2003	8.12	2011	2013	▂▂▂▂▂▂▂▂ ▃▃▃ ▂▂▂▂▂▂▂▂▂▂▂▂▂
Multiple fungal pathogen	2003	8.43	2014	2018	▂▂▂▂▂▂▂▂▂▂▂ ▃▃▃▃▃ ▂▂▂▂▂▂▂▂
Linkage map	2003	8.02	2015	2017	▂▂▂▂▂▂▂▂▂▂▂▂ ▃▃▃ ▂▂▂▂▂▂▂▂▂
Immunity	2003	9.89	2019	2021	▂▂▂▂▂▂▂▂▂▂▂▂▂▂▂▂▂▂▂▂▂ ▃▃▃
Influence	2003	9.50	2019	2021	▂▂▂▂▂▂▂▂▂▂▂▂▂▂▂▂▂▂▂▂▂ ▃▃▃
Rhizosphere microorganism	2003	8.58	2019	2021	▂▂▂▂▂▂▂▂▂▂▂▂▂▂▂▂▂▂▂▂▂ ▃▃▃
Bacterial community	2003	8.58	2019	2021	▂▂▂▂▂▂▂▂▂▂▂▂▂▂▂▂▂▂▂▂▂ ▃▃▃
Spp.	2003	8.27	2019	2021	▂▂▂▂▂▂▂▂▂▂▂▂▂▂▂▂▂▂▂▂▂ ▃▃▃
Quantitative	2003	8.07	2019	2021	▂▂▂▂▂▂▂▂▂▂▂▂▂▂▂▂▂▂▂▂▂ ▃▃▃

## Data Availability

Not applicable.

## Supplementary Materials

The following supporting information can be downloaded at: https://www.mdpi.com/article/10.3390/toxics10100624/s1, Table S1: Number of publications by countries. Table S2: Number of documents issued by the organizations. Table S3: Number of articles published in journals. Table S4: Number of articles published by the author. Table S5: Main keyword occurrence statistics.
